# Tungsten oxide supported on copper ferrite: a novel magnetic acid heterogeneous catalyst for biodiesel production from low quality feedstock†

**DOI:** 10.1039/d2ra06923g

**Published:** 2022-12-02

**Authors:** Hiarla Cristina Lima dos Santos, Matheus Arrais Gonçalves, Alexandre da Cas Viegas, Bruno Apolo Miranda Figueira, Patrícia Teresa Souza da Luz, Geraldo Narciso da Rocha Filho, Leyvison Rafael Vieira da Conceição

**Affiliations:** Federal University of Pará, Institute of Exact and Natural Sciences, Graduate in Chemistry Program, Laboratory of Catalysis and Oleochemical 66075–110 Belém Pará Brazil rafaelvieira@ufpa.br +55 91 98102 1185; Federal University of Rio Grande do Sul, Institute of Physics 90035–190 Porto Alegre Rio Grande do Sul Brazil; Federal University Western Pará, Graduate in Environmental Society and Quality of Life 68040–255 Santarém Pará Brazil; Federal Institute of Education, Science and Technology of Pará, Department of Education, Science and Teacher Education 66093–020 Belém Pará Brazil

## Abstract

This study aims to synthesize a WO_3_/CuFe_2_O_4_ catalyst through a wet impregnation method and use it as a new magnetic acid catalyst in the transesterification process of waste cooking oil (WCO). The results of the characterization by XRD, FTIR, SEM, EDS, TG/DTG, VSM and Surface Acidity showed that the obtained bifunctional catalyst has been successfully synthesized. The study of the reaction parameters, such as reaction temperature (140–180 °C), reaction time (1–5 h), molar ratio MeOH : oil (25 : 1–45 : 1) and catalyst loading (2–10% m m^−1^) was performed in the conversion of WCO into biodiesel *via* transesterification. The reactional behavior showed the following optimal reaction conditions: reaction temperature of 180 °C, reaction time of 3 h, molar ratio MeOH : oil of 45 : 1 and catalyst loading of 6%. Based on the results, biodiesel with a maximum ester content of 95.2% was obtained using the WO_3_/CuFe_2_O_4_ magnetic catalyst under the optimal reaction conditions. The magnetic catalyst showed excellent catalytic and magnetic performance and it was applied in five reaction cycles with ester content above 80%. Biodiesel properties were found in accordance with ASTM limits. This research provided the development of a stable and reusable WO_3_/CuFe_2_O_4_ bifunctional catalyst for potential application in biodiesel production.

## Abstract 2

This study aims to synthesize a WO_3_/CuFe_2_O_4_ catalyst through a wet impregnation method and use it as a new magnetic acid catalyst in the transesterification process of waste cooking oil (WCO).

## Graphical abstract

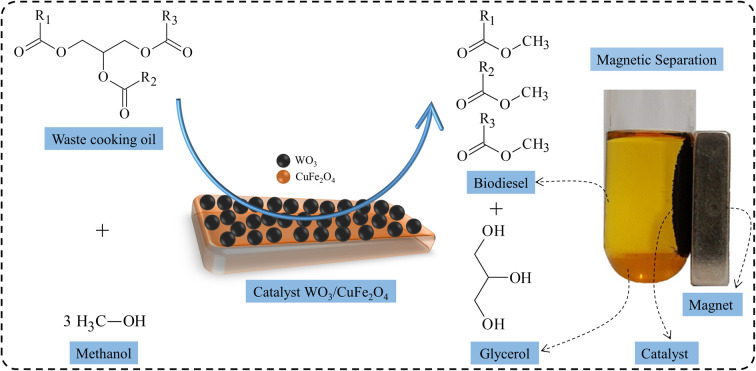

## 1. Introduction

In recent decades, new energy sources that are renewable and less polluting have been researched and compared to fossil fuels, widely used today.^1,2^ In this sense, biodiesel emerges as one of the most promising energy alternatives, since it is a renewable, biodegradable and considerably less polluting fuel than petroleum diesel.^3,4^ Chemically, biodiesel could be defined as alkyl esters, produced through the transesterification and esterification reactions of triacylglycerols in the presence of short-chain alcohols, such as methanol and ethanol.^4^

In the literature, several sources of triacylglycerol have been applied in fuels production, edible and inedible oils, algae, *etc.*^5–7^ In addition, the use of residual oilseed matrices has been studied in order to reduce biodiesel production costs, since the raw materials used in biodiesel production represent about 60–75% of the process total cost.^8,9^ In that regard, biodiesel production is reported in the literature through the use of residual sources of triglycerides, such as animal fats, waste cooking oils, *etc.*^9,10^

Transesterification and esterification reactions are usually performed in the presence of homogeneous, heterogeneous or enzymatic catalysts.^11,12^ Heterogeneous catalysts have been attracting attention due to the possibility of being easily recovered and reused in more than one reaction cycle, which results in the reduction of the biodiesel production process final cost.^12,13^ The main types of heterogeneous catalysts applied to synthesis of compounds with high value added, such as biodiesel, reported in the literature are: zeolites, biochar carbons, mesoporous silicas, metal oxides, *etc.*^14–19^

In addition, among the class of heterogeneous catalysts, the catalysts with acidic properties stand out because it is possible to apply them, in esterification and transesterification reactions, in the presence of residual raw materials without causing inconveniences, such as soap production, reactor corrosion, *etc.*^12,20,21^ In this sense, the use of metal oxides, such as tungsten oxide (WO_3_), in biodiesel production is reported in the literature due to its strong acidity of Brønsted and Lewis.^22^ The work developed by Xie and Yang studied the application of a catalyst composed of WO_3_ supported in AlPO_4_ in the production of biodiesel from soybean oil. The results presented a ester conversion of 72.5% in the following reaction conditions: reaction temperature of 180 °C, MeOH : oil molar ratio of 30 : 1, catalyst loading of 5% and reaction time of 5 h.^23^

Among the heterogeneous catalysts magnetic materials have been attracting attention due to the fact that their magnetic properties facilitate the separation step of the catalyst from the reaction medium (usually using techniques such as filtration and centrifugation), through the application of a magnetic field, which would reduce the costs employed in the separation stage and consequently the total costs of the process.^24,25^ Several materials have been applied as catalysts or catalytic supports in the production of biodiesel, such as magnetites (Fe_3_O_4_), hematites (Fe_2_O_3_) and ferrites (MFe_2_O_4_), where M is the transition metal.^25,26^

Seffati *et al.* studied biodiesel production through the use of chicken fat and methanol. The catalyst used in the reaction consisted of the impregnation of calcium oxide (CaO) in copper ferrite (CuFe_2_O_4_) and the biodiesel obtained ester content of 94.52% in the following optimal reaction conditions: reaction temperature of 70 °C, MeOH : oil molar ratio of 15 : 1, 3% catalyst loading and reaction time of 4 h.^8^

This study aims to study the application of a heterogeneous magnetic acid catalyst, composed of WO_3_ impregnated in cooper ferrite (CuFe_2_O_4_), in the production of biodiesel using waste cooking oil and methanol. The effects of the variables present in the transesterification reaction were investigated in the catalytic activity of the catalyst, such as reaction temperature, reaction time, molar ratio MeOH : oil and catalyst loading. It is noteworthy that biodiesel synthesis by magnetic acid catalyst using CuFe_2_O_4_ as catalytic support has not been reported in the literature yet.

## 2. Experimental

### 2.1. Materials

All reagents used were analytical. Copper acetate II (CuC_4_H_6_O_4_·H_2_O, Dinâmica®, 98%) and iron nitrate (iii) (Fe(NO_3_)_3_·9H_2_O, Neon®, 98%) were used as precursors in the synthesis of CuFe_2_O_4_. Dihydrate sodium tungstate (Na_2_WO_4_·2H_2_O, Scientific exodus®, 99%) was used in the catalyst preparation stage. Sodium hydroxide (NaOH, Neon®, 97%) and hydrochloric acid (HCl, Isofar®, 37%) were used to determine the surface acidity of the catalyst. Methyl alcohol (CH_3_OH, Dinâmica®, 99.8%) and waste cooking oil (WCO), collected from the restaurant of the Federal University of Pará, were used in the transesterification reaction. Methyl heptadecanoate (C_18_H_36_O_2_, Sigma-Aldrich, 99%) and heptane (C_7_H_16_, Dynamics®, 99.5%) were used in chromatographic analysis. Ethanol (C_2_H_5_OH, Scientific exodus®, 99.8%) was used in the catalyst washing process. Table 1 shows the fatty acid composition and the physical–chemical properties of WCO.

**Table 1:** Fatty acid composition and physicochemical properties of WCO used to produce biodiesel

Properties	Value
**Fatty acid composition, wt (%)**	
Palmitic (C16 : 0)	12.0
Stearic (C18 : 0)	4.2
Oleic (C18 : 1)	24.5
Linoleic (C18 : 2)	51.0
Linolenic (C18 : 3)	6.2
Others	2.1

**Physicochemical properties**	
Acid value, (mg KOH g^−1^) (AOCS Cd 3d–63)	4.1
Saponification value, (mg KOH g^−1^) (AOCS Tl 1a–64)	187.1
Viscosity at 40 °C, (mm^2^ s^−1^)	36.4
Moisture content, (%) (AOCS Ca 2b–38)	0.2
Molecular weight, (g mol^−1^)	864

### 2.2. Preparation of heterogeneous magnetic acid catalyst (WO_3_/CuFe_2_O_4_)

#### 2.2.1. Synthesis of copper ferrite (CuFe_2_O_4_)

Copper ferrite (CuFe_2_O_4_) was prepared using the coprecipitation method according to the methodology adapted by Seffati *et al.*^8^ Initially, the required amount of CuC_4_H_6_O_4_·H_2_O and Fe(NO_3_)_3_·9H_2_O at molar ratio 1 : 2 (Cu : Fe) were dissolved into 150 mL of distilled water followed by mechanical agitation at room temperature during 30 min. Then, a solution of NaOH 4 mol L^−1^ was added drop by drop to adjust the pH of the mixture to 12. At the end of the precipitation process, the system was kept under mechanical agitation at 65 °C for 4 h. Thus, the obtained product was washed with distilled water several times until the washing water achieve neutral pH (pH = 7), and finally, the material was dried in an oven at 80 °C for 12 h and calcined at 500 °C for 3 h (10 °C min^−1^) in order to obtain the CuFe_2_O_4_.

#### 2.2.2. Impregnation of the active phase

**Fig. 1:**
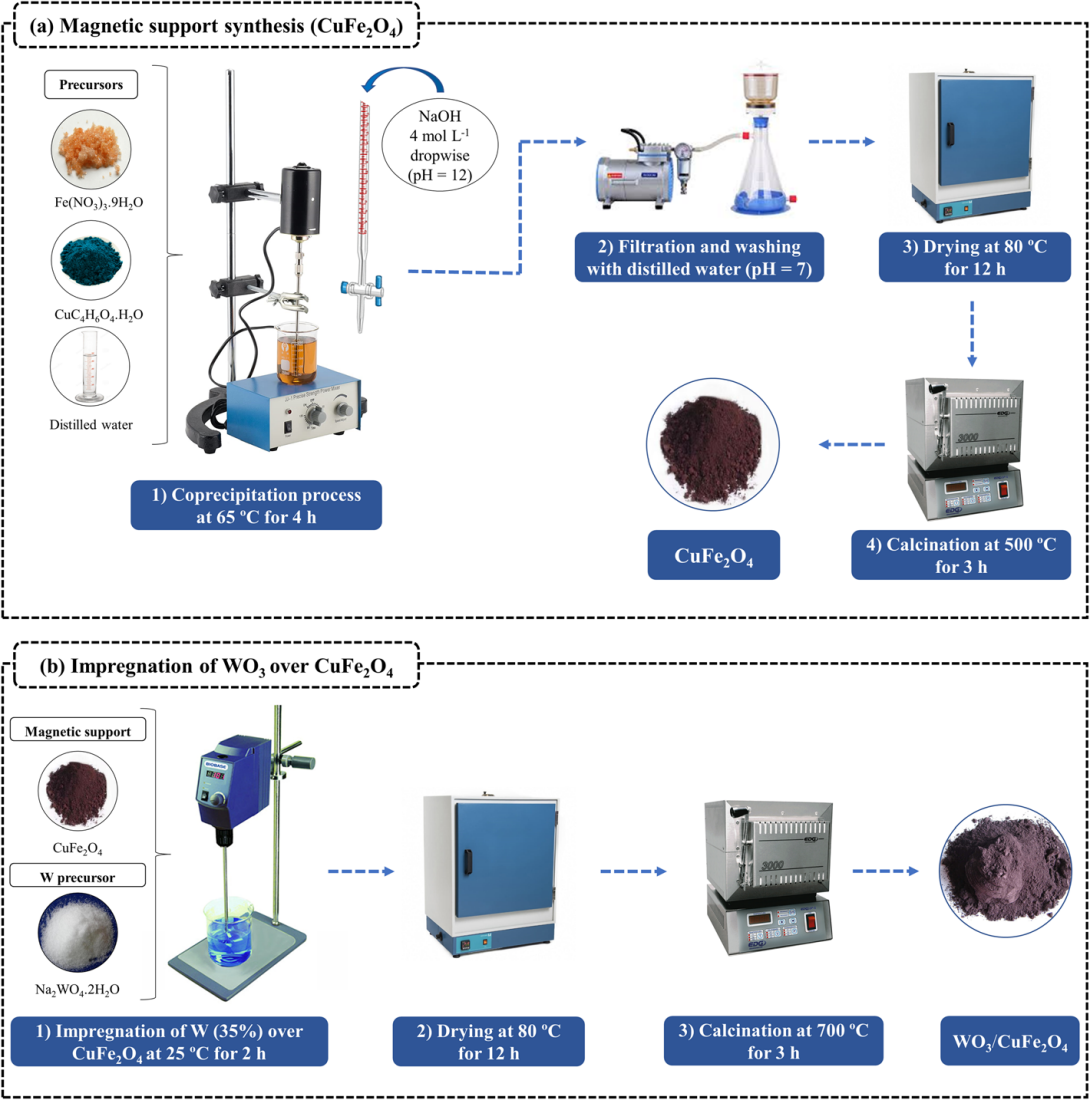
Schematic diagram of WO_3_/CuFe_2_O_4_ bifunctional heterogeneous catalyst synthesis.

In the process of synthesis of the magnetic acid catalyst, the method of wet impregnation was applied by using the same tungsten precursor and calcination temperature previously described by Kaur *et al.*^27^ A series of WO_3_ impregnated CuFe_2_O_4_ catalysts was prepared (20–40% WO_3_ loading), where the catalyst with 35% of WO_3_ on the support was chosen as the best catalyst (Fig. S1, see ESI†). In a typical procedure, approximately 0.97 g of Na_2_WO_4_·2H_2_O was dispersed in 10 mL of distilled water to obtain 35% of the metal W on the surface of the support. Then 1.0 g of CuFe_2_O_4_ was added to the system. The mixture was kept under constant mechanical agitation for 2 h at room temperature. Then, the material was dried in an oven at 80 °C/12 h and calcined at 700 °C/3 h (10 °C min^−1^). Fig. 1 illustrates the schematic diagram of the catalyst preparation, designated as WO_3_/CuFe_2_O_4_.

### 2.3. Catalyst characterization techniques

The materials were characterized by several techniques such as X-Ray Diffraction (XRD): Bruker diffractometer, model D2 PHASER, Cu radiation (Kα = 1.54 Å), 40 kV, 30 mA, 5° < 2*θ* > 75° analysis range. Fourier Transform Infrared Spectroscopy (FTIR): Prestige 21 Model Shimadzu Spectrometer, spectral range of analysis was 2500–400 cm^−1^, resolution of 4 cm^−1^ and 32 scans. Scanning Electron Microscopy (SEM): Tescan microscope, Vega 3 LMU model. Energy Dispersion X-Ray Spectroscopy (EDS): Oxford micro-analysis system, AZTec Energy X-Act model, resolution 129 eV. Thermogravimetric Analysis (TG/DTG): Shimadzu equipment, model DTG-60H, temperature range from 25 to 800 °C (heating rate of 10 °C min^−1^), nitrogen flow of 50 mL min^−1^, alumina crucible. Vibrant Sample Magnetometry (VSM): Microsense magnetometer, EZ9 model, room temperature, applied magnetic field from −20 000 Oe to 20 000 Oe. Surface Acidity: a mass sample of 0.1 g was placed into 20 mL of standardized NaOH 0.1 mol L^−1^ solution, remaining under agitation for 24 h at room temperature. Subsequently, the sample was centrifuged and the supernatant was tilted with standardized HCl 0.1 mol L^−1^ solution in the presence of phenolphthalein as an indicator.^28^

### 2.4. Transesterification reaction

The reactions were performed in a PARR 5000 Multireactor reactor with fixed agitation at 700 rpm, the following reaction conditions were investigated: reaction temperature (140–180 °C); reaction time (1–5 h); MeOH : oil molar ratio (25 : 1–45 : 1) and catalyst loading (2–10% m m^−1^). After the reaction, the catalyst was separated by the application of the external magnetic field to the reaction system. The reaction products were transferred to a funnel, separated and washed with 500 mL of distilled water (60 °C) for removal of residual alcohol and glycerol. Finally, the biodiesel samples were stored for further analysis.

### 2.5. Determination of biodiesel properties

The methyl ester content of biodiesel samples was determined by gas chromatography according to the methodology adapted from the European standard (EN 14103) proposed by Silva *et al.*^29^ In this method, the Varian gas chromatograph, model CP 3800, equipped with Flame Ionization Detector (FID) and capillary column CP WAX 52 CB (30 m long, 0.32 mm in diameter and 0.25 μm film) was used. Methyl heptadecanoate was used as an internal standard and heptane as a solvent. In addition, the oven temperature programming from 170 °C to 250 °C (same FID temperature) was used at a rate of 10 °C min^−1^, helium gas was used as a mobile phase with flow of 1.0 mL min^−1^, and the injection volume was 1 μL of sample. The ester content (EC) was calculated according to eqn (1):1
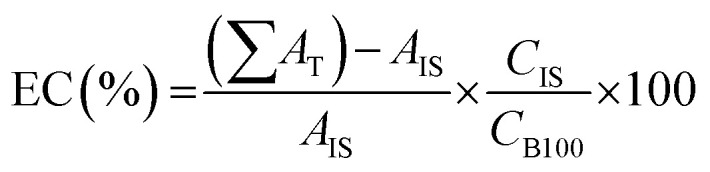
where: ∑*A*_T_ is the sum of the peaks total area; *A*_IS_ is the peak area of the internal pattern; *C*_IS_ is the solution concentration of the internal standard (mg L^−1^); *C*_B100_ is biodiesel's concentration after dilution (mg L^−1^).

The main physicochemical properties of biodiesel were determined by a standard method of the American Society for Testing and Materials (ASTM). The kinematic viscosities, analyzed at 40 °C, were determined for the biodiesel samples synthesized according to the ASTM D445 method, using a viscometer model Cannon-Fenske (SCHOTT GERATE, 520 23). The density, analyzed at 20 °C, was measured by ASTM D6890 method on a KEM DAS-500 automatic densimeter. The acid value was evaluated conforming to the ASTM D664 method. The flash point was estimated employing the ASTM D093 method on an automatic TANAKA APM 7 Pensky-Martens flash point. The cold filter plugging point was determined using ASTM D6371 methodology on a TANAKA equipment AFP-102 model. The corrosiveness to copper was appraised by ASTM D130 in a copper corrosion bath from Koehler. The oxidative stability was determined using a Rancimat, model 743 from Metrohm, in accordance with EN 14112.

### 2.6. Reuse study

The reuse study was conducted for the magnetic catalyst under optimal transesterification reaction conditions. After each reaction cycle, the catalyst was separated from the reaction medium by the application of the external magnetic field, washed once with heptane (10 mL) and twice with ethyl alcohol (25 mL) and dried in an oven at 80 °C for 12 h.

The process of recalcination of the magnetic catalyst was proposed by observing the decrease of the ester content values during the reaction cycles. This study was conducted in two stages. First, the catalyst was reproduced and washed after each reaction cycle, as described above. Then, the catalyst was thermally reactivated by calcination at 500 °C for 3 h, in a muffle oven, to eliminate organic components from the surface of the material.

## 3. Results and discussion

### 3.1. Physical–chemical evaluation of the magnetic catalyst

The CuFe_2_O_4_ support and WO_3_/CuFe_2_O_4_ magnetic catalyst were characterized by XRD, FTIR, SEM/EDS, TGA/DTGA, VSM and Surface Acidity techniques.

#### 3.1.1. XRD analysis

**Fig. 2:**
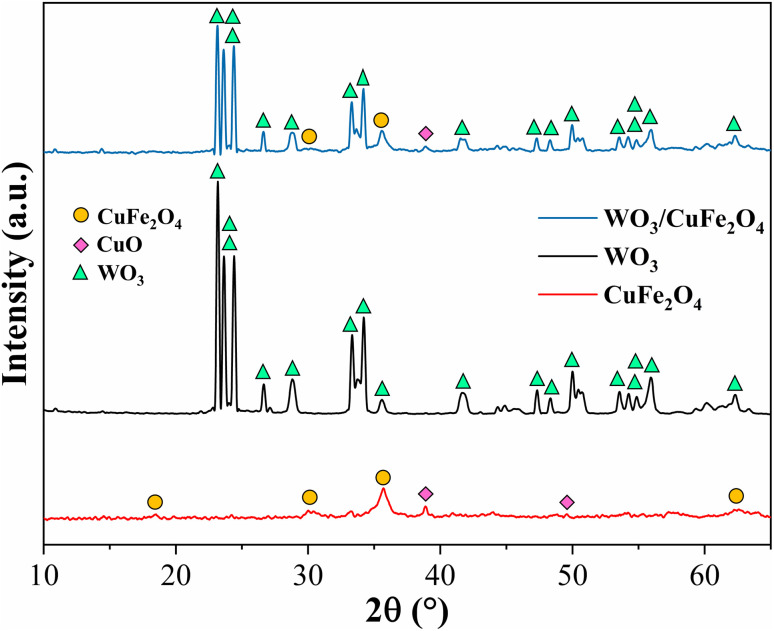
XRD patterns of CuFe_2_O_4_, WO_3_ and WO_3_/CuFe_2_O_4_ catalyst.

The X-ray diffraction patterns of CuFe_2_O_4_, WO_3_ and WO_3_/CuFe_2_O_4_ materials are presented in Fig. 2. The material corresponding to the CuFe_2_O_4_ phase (red line) exhibited peaks at 2*θ* = 18.44°, 29.98°, 35.67° and 62.49°, which were well correlated to crystallographic planes (111), (220), (311) and (440), with cubic system and spatial group *Fd̄*3*m* (ICDD 01-077-0010), confirming the formation of CuFe_2_O_4_ with reverse spinel structure, in which Fe^3+^ ions are situated in an equivalent way in the tetrahedral and octahedral sites, Cu^2+^ is only in octahedral sites.^30,31^ In addition, the diffractogram also showed peaks at 2*θ* = 38.87° and 48.95° corresponding to the planes (111) and (−202) of CuO with monoclinic system (ICDD 03-065-2309). According to Nikolić, the formation of this impurity may be related to the oxidation of Cu^2+^ ions during the synthesis process carried out under atmospheric conditions.^32^

For the sample containing the crystalline phase WO_3_ (black line), the presence of well-formed peaks with high intensity was identified in 2*θ* = 23.18°, 23.64°, 24.45°, 26.65°, 28.70°, 33.30° and 34.20°, which were directly indexed to crystallographic planes (001), (020), (200), (120), (111), (021) and (220) of this oxide with monoclinic system (ICDD 01-075-2072).^27^ The presence of phases WO_3_ and CuFe_2_O_4_ (blue line) in the magnetic catalyst was confirmed in the XRD pattern, whose reduction in intensity of the main peaks revealed the probable dispersion of WO_3_ in CuFe_2_O_4_. Additionally, the presence of peaks in 2*θ* = 29.98° and 25.67° was identified, which were well indexed to the CuFe_2_O_4_ phase and 2*θ* = 38.87° to the CuO phase, both phases derived from the magnetic support CuFe_2_O_4_.

#### 3.1.2. FTIR analysis

**Fig. 3:**
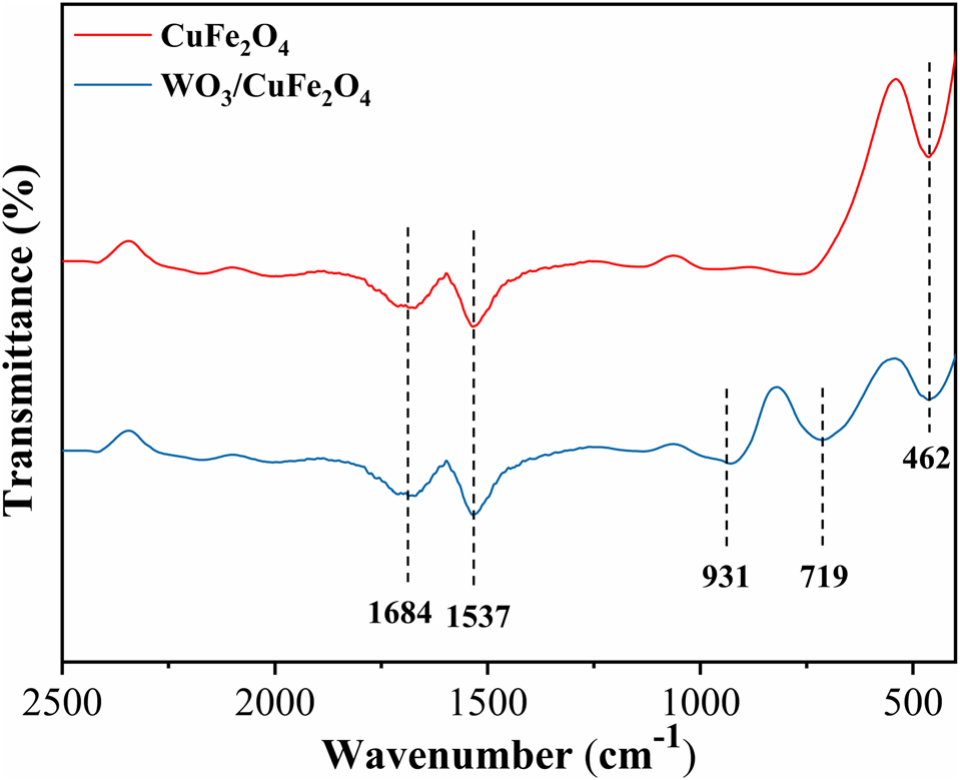
FTIR spectra of the CuFe_2_O_4_ support and WO_3_/CuFe_2_O_4_ catalyst.

The FTIR spectra of the CuFe_2_O_4_ magnetic support and the WO_3_/CuFe_2_O_4_ bifunctional catalyst are presented in Fig. 3. The spectrum referring to CuFe_2_O_4_ indicated absorption bands around 462, 1537 and 1684 cm^−1^. The band at 462 cm^−1^ is attributed to metal–oxygen elongation (M–O, M = Cu or Fe), specific to spinel ferrite.^8^ The bands in 1537 and 1684 cm^−1^ are related to the stretching vibration O–H and the deformation of the group –OH, respectively, and the absorption of H_2_O on the metal surface.^33^ Moreover, all CuFe_2_O_4_ spectrum absorptions are present in the WO_3_/CuFe_2_O_4_ magnetic catalyst spectrum, however, new absorption vibrations occur at 719 and 931 cm^−1^ referring to the stretch bands of the O–W–O and W=O connections, respectively.^34,35^ Thus, the analysis of the data from the FTIR spectra presented suggest the efficiency of the ferrite synthesis process and the impregnation of the WO_3_ species in the magnetic support to obtain the bifunctional character of the catalyst.

#### 3.1.3. SEM and EDS analysis

**Fig. 4:**
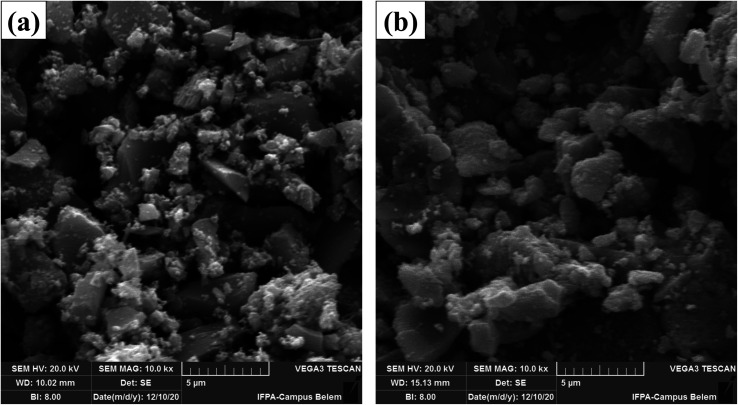
SEM micrographs with 10 000-fold magnification of (a) CuFe_2_O_4_ magnetic support and (b) WO_3_/CuFe_2_O_4_ magnetic catalyst.

The surface morphology of the magnetic materials CuFe_2_O_4_ and WO_3_/CuFe_2_O_4_ was examined by SEM analysis (Fig. 4). The SEM micrographs of the CuFe_2_O_4_ magnetic support and WO_3_/CuFe_2_O_4_ magnetic catalyst revealed that both materials have structures composed of particle clusters of different shapes and sizes (≤5 μm). The interaction between magnetic particles is responsible for the morphological nature (clusters) of these materials as reported in previous studies.^36^ The SEM micrograph referring to CuFe_2_O_4_ (Fig. 4a), evidences the rough and spongy appearance in the analyzed region. It is noteworthy that this characteristic is also reported in the study of nanocatalyst synthesis based on CuFe_2_O_4_ developed by Rajput *et al.*^37^ In addition, when analyzing the SEM micrograph of the WO_3_/CuFe_2_O_4_ catalyst (Fig. 4b), the disappearance of the spongy aspect is observed, this may be related to the dispersion of the WO_3_ phase over the support.

**Fig. 5:**
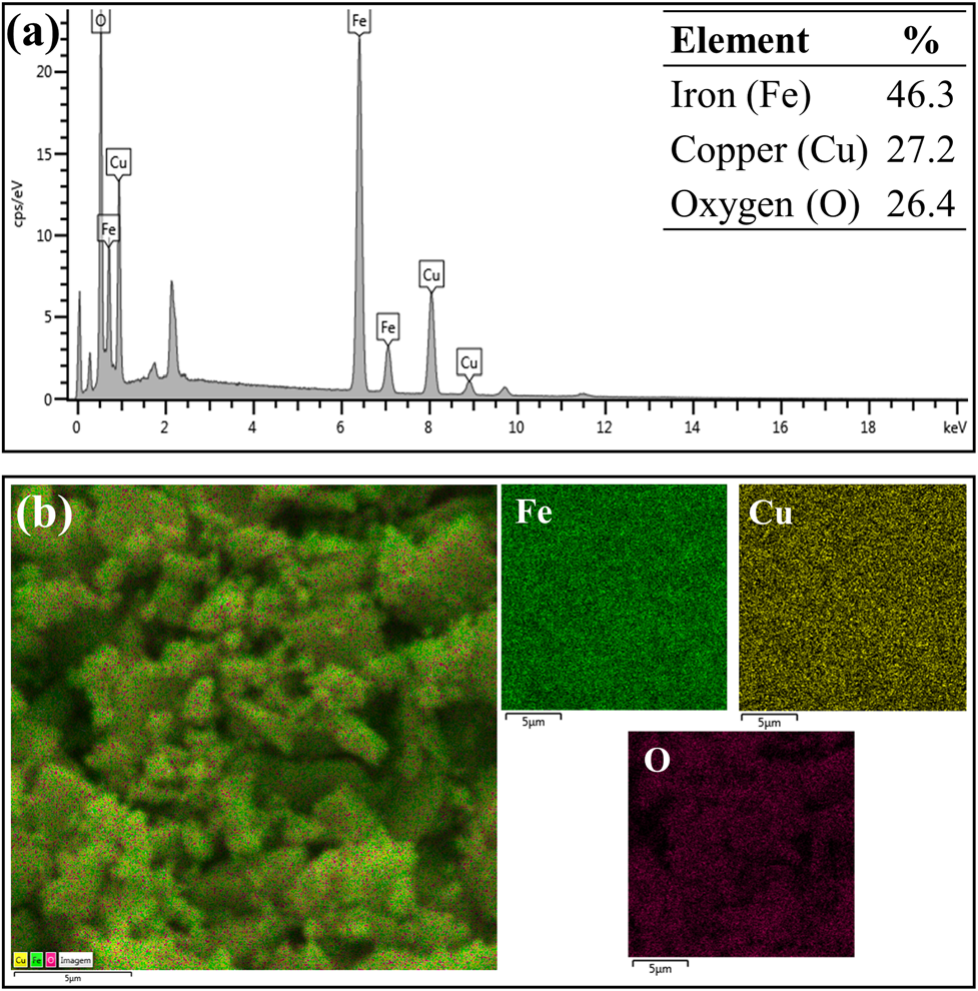
EDS analysis (a) chemical composition of the CuFe_2_O_4_ and (b) elemental map of each chemical element on the surface of the CuFe_2_O_4_.

**Fig. 6:**
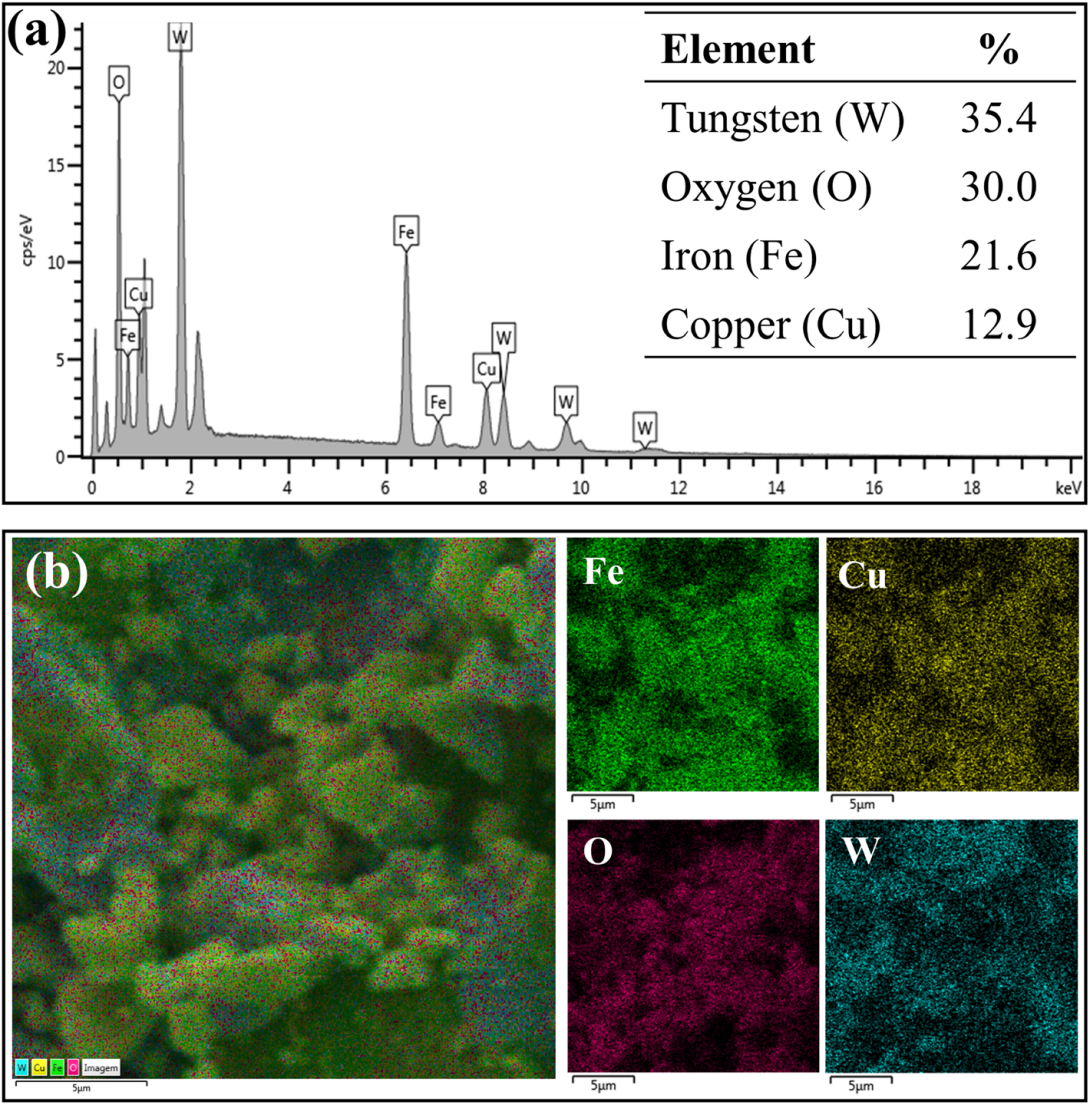
EDS analysis (a) chemical composition of the WO_3_/CuFe_2_O_4_ catalyst and (b) elemental map of each chemical element on the surface of the WO_3_/CuFe_2_O_4_ catalyst.

Fig. 5 and 6 show EDS spectroscopy data that were collected in the selected region of micrographs shown in Fig. 4. Fig. 5 presents the results obtained by chemical composition EDS and elementary surface mapping of the CuFe_2_O_4_ support. Fig. 5a displays the EDS spectrum of CuFe_2_O_4_, which demonstrates the main peaks corresponding to the elements iron (Fe), copper (Cu) and oxygen (O) present on the surface of the magnetic support at percentage concentrations (m m^−1^) of 46.3%, 27.2% and 26.4%, respectively. The elemental composition presented Fe/Cu ratio of 1.7, a value relatively close to that desired in the synthesis of CuFe_2_O_4_ (Fe/Cu = 2.0). The copper ferrite presented the elements evenly distributed on the surface of the material, as shown by the elemental surface mapping in Fig. 5b, indicating the efficiency of the used synthesis process by coprecipitation.

Fig. 6a represents the results of elemental composition of the WO_3_/CuFe_2_O_4_ catalyst. In the analysis, the presence of the tungsten element (W) on the surface of the catalytic material was verified, in addition to the characteristic elements of CuFe_2_O_4_, corroborating the XRD and FTIR analysis data presented earlier. The W element content of 35.4% (value close to theoretically stipulated) shows the efficiency of the impregnation process, while the increase in the content of element O, in about 26.0% (ferrite) to 30.0% (catalyst), indicates the formation of the species WO_3_. According to Fig. 6b, it is possible to verify that element W is relatively well dispersed on the surface of the catalytic support, and this uniform distribution of W is essential for the catalytic activity of the magnetic catalyst developed.

#### 3.1.4. TG/DTG analysis

**Fig. 7:**
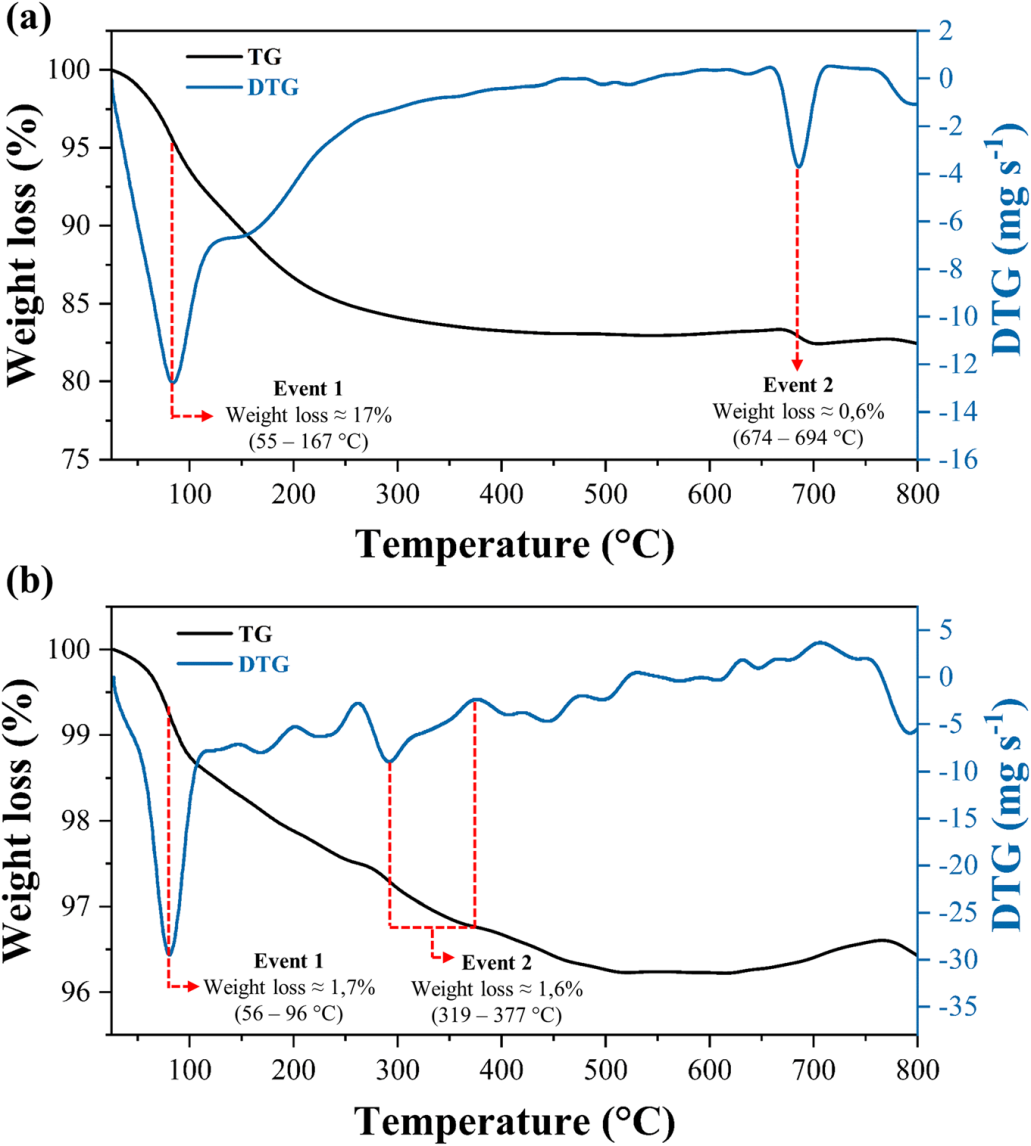
TG/DTG plots of (a) CuFe_2_O_4_ support and (b) WO_3_/CuFe_2_O_4_ catalyst.

Fig. 7 illustrates the thermogravimetric profiles of CuFe_2_O_4_ and catalyst WO_3_/CuFe_2_O_4_ non-calcined. The results showed two main stages of mass loss for the materials CuFe_2_O_4_ and WO_3_/CuFe_2_O_4_. For the CuFe_2_O_4_ magnetic support (Fig. 7a), the first stage of thermal decomposition occurs in the temperature range of 55–167 °C, corresponding to the mass loss of 17%, which is associated with the removal of physically adsorbed water and hydroxyl groups on the surface of the material, in addition to the decomposition of organic components such as the AcO^−^ ion (acetate) from the metallic precursor.^38,39^ A small mass loss, 0.6%, was observed in the temperature range of 674–694 °C, and may be related to the phase transition from tetragonal structure to cubic structure, indicating the development of a more stable phase for ferrite CuFe_2_O_4_.^38,40^ However, the heat treatment at 500 °C made the material CuFe_2_O_4_ stable.

The TG curve of the WO_3_/CuFe_2_O_4_ catalyst (Fig. 7b) shows the first mass loss event of 1.7% around 56–96 °C referring to the structural water loss of the material surface. The increase in temperature leads to a reduction in material mass of approximately 1.6% in the range of 319–377 °C, due to the formation of the crystalline phase of WO_3_ of monoclinic structure, becoming stable above 377 °C.^41^ Thus, the results showed that the WO_3_/CuFe_2_O_4_ catalyst has greater thermal stability when compared to the magnetic support CuFe_2_O_4_.

#### 3.1.5. VSM analysis

**Fig. 8:**
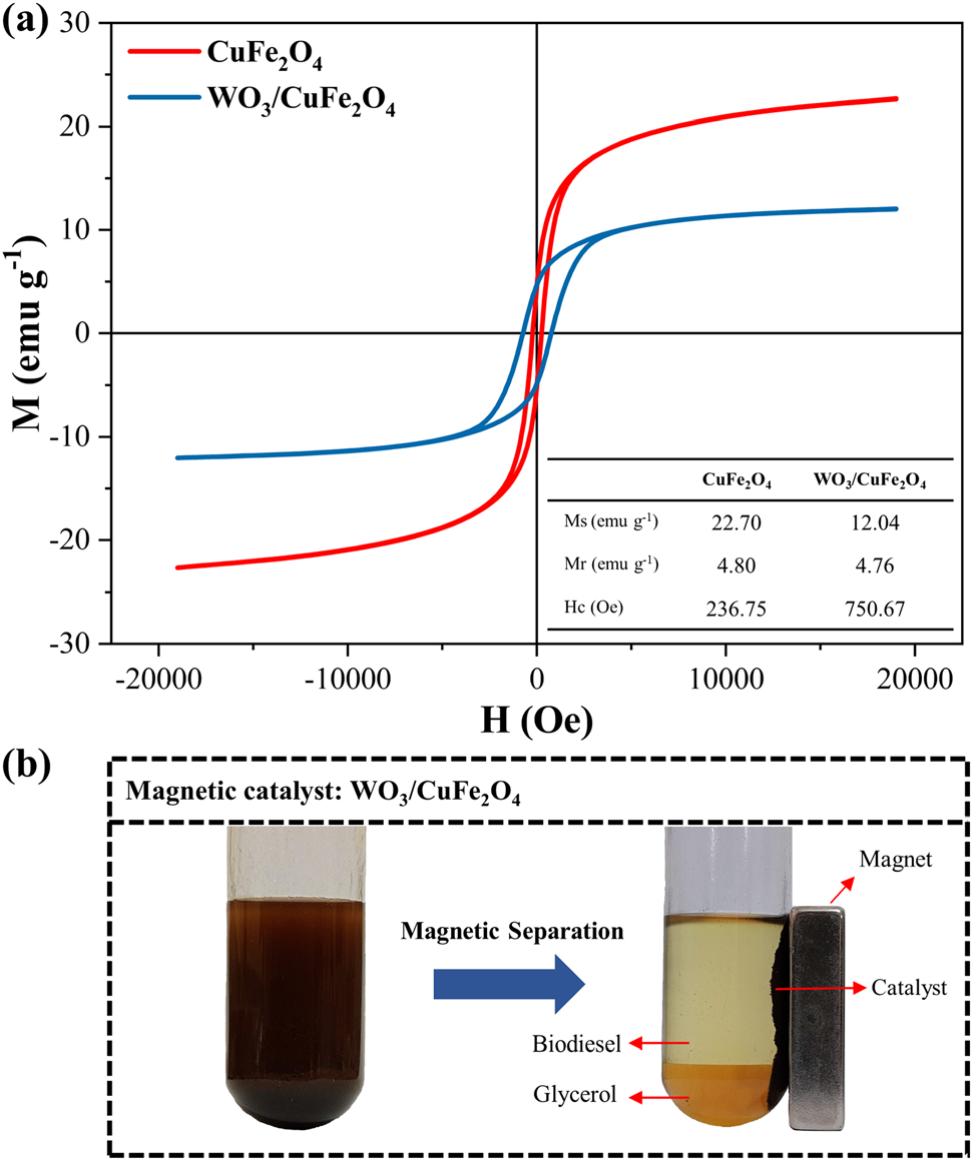
(a) *M*–*H* curve of CuFe_2_O_4_ support and WO_3_/CuFe_2_O_4_ catalyst; (b) Illustration of the magnetic separation process of the WO_3_/CuFe_2_O_4_ catalyst in the transesterification reaction.

The magnetic behavior of CuFe_2_O_4_ and WO_3_/CuFe_2_O_4_ materials is described by *M*–*H* hysteresis curves (magnetization *vs.* applied magnetic field) obtained by VSM analysis (Fig. 8a).

The magnetic properties investigated were saturation magnetization (*M*_s_), remaining magnetization (*M*_r_) and coercivity (*H*_c_). Based on the results obtained, the CuFe_2_O_4_ support (red curve) and the WO_3_/CuFe_2_O_4_ catalyst (blue curve) showed saturation magnetization values of 22.70 emu g^−1^ and 12.04 emu g^−1^, respectively, when a field of ±20 000 Oe was applied at room temperature. It is observed that the *M*_s_ value of the WO_3_/CuFe_2_O_4_ catalyst decreased in relation to CuFe_2_O_4_ due to the incorporation of the non-magnetic species WO_3_ in the magnetic support structure, causing a 46.9% reduction in the magnetic activity of the catalyst. However, catalyst magnetization remains effective for the separation process. According to the literature, magnetic catalysts such as CaFe_2_O_4_–CaFe_2_O_5_–Fe_3_O_4_, MgO/MgAl_0.4_Fe_1.6_O_4_ and CaO@Sr_2_Fe_2_O_5_–Fe_2_O_3_ were applied in the transesterification process and presented *M*_s_ values at magnitudes 0.217 emu g^−1^, 2.02 emu g^−1^and 11.09 emu g^−1^, respectively.^42–44^ In addition, the hysteresis *M*–*H* graph shows the curves in the regular sigmoidal format typical of CuFe_2_O_4_, and presents *M*_r_ and *H*_c_ values equal to 4.80 emu g^−1^ and 236.75 Oe, respectively. These results classify the ferrite synthesized in this study as mild ferromagnetic and indicate a certain resistance to demagnetizations.^45,46^ The WO_3_/CuFe_2_O_4_ catalyst also demonstrated ferromagnetic behavior and a higher resistance to demagnetizations by presenting *M*_r_ and *H*_c_ values equal to 4.76 emu g^−1^ and 750.67 Oe, respectively.

The arrangement of the WO_3_/CuFe_2_O_4_ catalyst in the system before and after magnetic separation, after the end of the reaction process, is presented in Fig. 8b. It is possible to observe that the magnetic properties of the developed catalyst are efficient to promote the process of separation and recovery of the catalyst from the reaction products (biodiesel and glycerol) when applied an external magnetic field, leading the process to full separation in a few minutes.

### 3.2. Influence of reaction parameters on biodiesel synthesis process

**Fig. 9:**
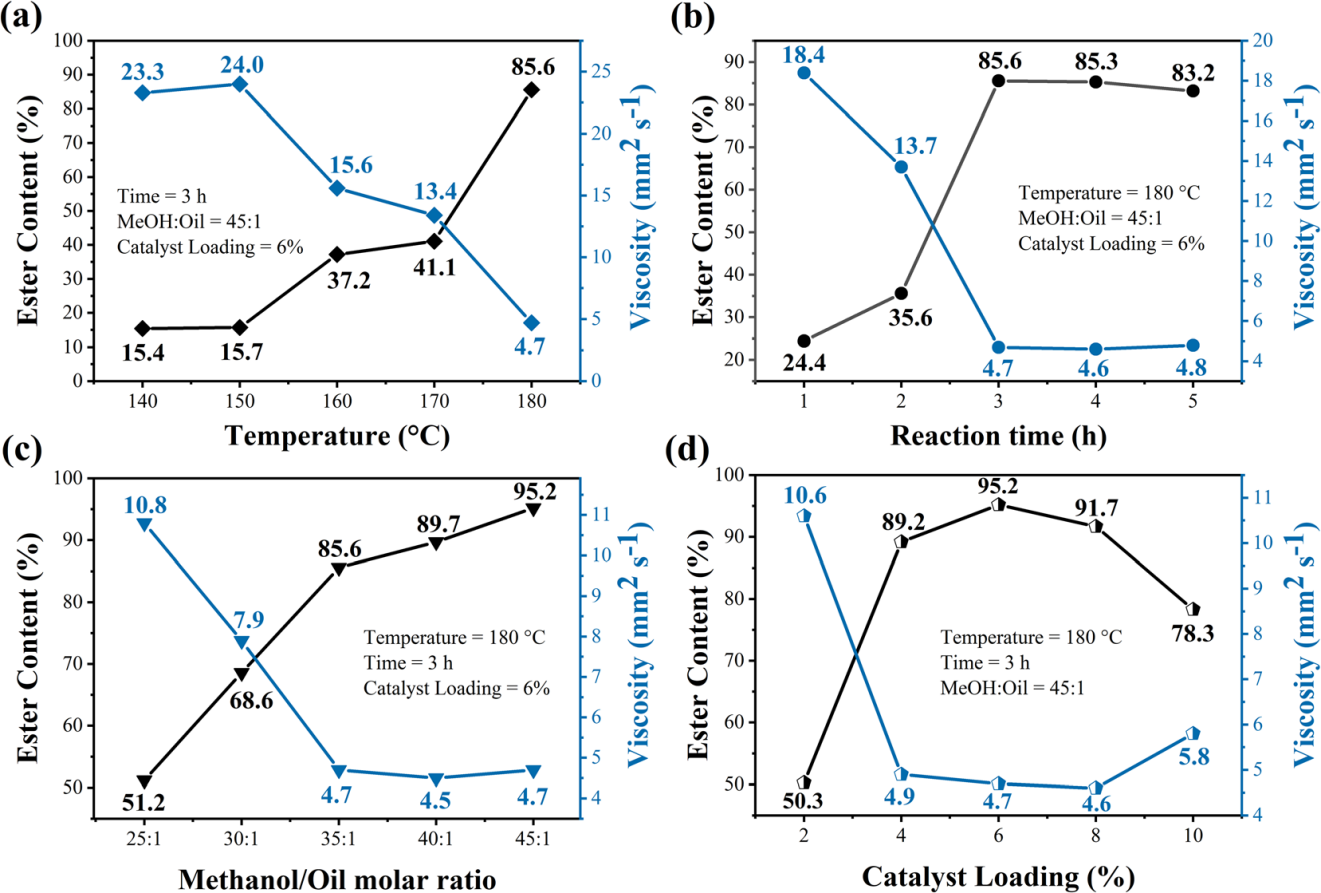
Investigation of the impact of (a) reaction temperature, (b) reaction time, (c) MeOH:oil molar ratio and (d) catalyst loading in the transesterification reaction using WO_3_/CuFe_2_O_4_ magnetic catalyst.

The biodiesel samples synthesized from different reactional conditions of reaction temperature, reaction time, MeOH : oil molar ratio and catalyst loading were evaluated for the ester content and kinematic viscosity, results presented in Fig. 9.

The effect of reaction temperature on the catalytic performance of the WO_3_/CuFe_2_O_4_ catalyst during the transesterification reaction was studied in the range from 140 to 180 °C (Fig. 9a). The highest ester conversion, 85.6%, was achieved for biodiesel synthesized at 180 °C. Thus, it is possible to infer that the transesterification process using the magnetic acid catalyst is strongly influenced by reaction temperature due to its endothermic nature. In general, reactions using acid catalysts require high temperatures due to low diffusion and reaction speed.^47^ Thus, the use of high temperatures has advantages to the reaction system, such as: increase in molecular collisions, kinetic energies and degrees of miscibility between the reagents. These factors favor the activation of the carbonyl group from the waste cooking oil triacylglycerols, allowing the nucleophilic attack of methanol, resulting in the production of methyl esters by the transesterification route.^33^ This influence of the temperature variable on the transesterification reaction is also reported in the studies developed by Jiménez-López *et al.* and Xie and Yang, in which they obtained biodiesel with ester contents of 92.0% and 72.5%, when the reactions were conducted at reaction temperatures of 200 °C and 180 °C, respectively.^23,48^

The kinematic viscosity of the biodiesel obtained suffered direct interference from the reaction temperature applied in the system. At a reaction temperature of 180 °C, the biodiesel obtained presents kinematic viscosity of 4.7 mm^2^ s^−1^. On the other hand, when the reaction was carried out at 140 °C, biodiesel presented a kinematic viscosity of 23.3 mm^2^ s^−1^. Thus, kinematic viscosity values tend to decrease with the increase of temperature. This behavior occurs due to the transfer of saturated chains to the biodiesel molecule as methyl esters are formed. Thus, there is a reduction of intermediates such as monoacylglycerol and diacylglycerol, compounds that may be responsible for increasing the viscosity of biodiesel.^49^

The study of the influence of time on the biodiesel synthesis process was carried out in the interval of 1 to 5 h. Based on the data presented on Fig. 9b, an increase in the value of the ester content for the biodiesel was observed from 24.4% to 85.6%, when the reactions are performed at reaction times from 1 to 3 h, respectively. However, the use of reaction times greater than 3 h did not presented significant changes in ester levels of biodiesel esters obtained, since biodiesel synthesized in 4 and 5 h resulted in ester contents of 85.3% and 83.2%, respectively. This slight decrease occurs due to the reversible nature of the transesterification reaction after reaching equilibrium.^50^ In addition, this influence is strongly observed in the kinematic viscosity of biodiesel synthesized in different reactional times, since there is a significant decrease in viscosity values from 18.4 mm^2^ s^−1^ to 4.7 mm^2^ s^−1^, when the reactions are processed at times from 1 and 3 h, respectively. Therefore, the time of 3 h was chosen as an optimal parameter for the transesterification reaction using the magnetic catalyst WO_3_/CuFe_2_O_4_.

Fig. 9c shows the impact of the variation of the molar ratio MeOH : oil from 25 : 1 to 45 : 1 on the transesterification reaction using the magnetic catalyst WO_3_/CuFe_2_O_4_. It is possible to observe from the results obtained that the catalytic efficiency of the WO_3_/CuFe_2_O_4_ catalyst increases as the MeOH : oil molar ratio is increased in the reaction, since the biodiesel synthesized using the MeOH : oil molar ratio of 25 : 1 presents an ester content of 51.2%, while the reaction performed in the MeOH : oil molar ratio of 45 : 1 leads to a biodiesel with an ester content of 95.2%, representing an increase of about 45% in the ester content. Thus, the MeOH : oil molar ratio of 45 : 1 was chosen as the most beneficial relationship for the process. In general, acidic nature catalysts require higher molar ratios to achieve a higher conversion into biodiesel, since a greater amount of methanol in the reaction medium favors the phenomena of mass transfer in the system and facilitates the access and performance of the catalyst to the substrate through the high internal pressure inside the closed reactor.^51,52^ The influence of the MeOH : oil molar ratio on the kinematic viscosity of biodiesel revealed a decrease in the values from 10.8 mm^2^ s^−1^ to 4.7 mm^2^ s^−1^ when using the MeOH : oil molar ratios of 25 : 1 and 45 : 1, respectively. It is noteworthy that a biodiesel with a kinematic viscosity value below 6.0 mm^2^ s^−1^ is desirable, as it facilitates the injection and dissolution of the fuel during its use.^53^

Catalyst loading is considered a key reaction parameter for the biodiesel production process. In order to evaluate the effect of the concentration of the magnetic catalyst WO_3_/CuFe_2_O_4_, catalytic tests were performed under the catalyst loading range of 2–10%. Fig. 9d shows that the efficiency of biodiesel production showed a significant improvement in the values of serum content from 50.3% to 95.2% when using catalyst loadings from 2 to 6%, respectively. This is due to the greater availability of active sites present in the reaction system, promoting greater contact of the oil–methanol–catalyst system.^54^ The use of catalyst loadings greater than 6% in the transesterification reaction causes a decrease in the ester content of the biodiesel, given that the use of 8 and 10% of catalyst in the process resulted in biodiesel with ester contents of 91.7 and 78.3%, respectively. This negative impact is related to mass transfer problems in the system due to excess catalyst, since a greater amount of catalyst increases the viscosity of the reaction mixture during the transesterification reaction of frying oil.^53^ The biodiesel obtained using the optimum catalyst loading condition of 6% showed kinematic viscosity of 4.7 mm^2^ s^−1^.

The results obtained in the study of influence of reaction variables applying the magnetic catalyst WO_3_/CuFe_2_O_4_, evidence the optimal reaction condition of the process: reaction temperature of 180 °C, reaction time of 3 h, molar ratio MeOH : oil of 45 : 1 and catalyst loading of 6%, which results in a biodiesel with a value of maximum ester content of 95.2%.

### 3.3. Physicochemical properties of biodiesel

Biodiesel obtained from waste cooking oil under optimal reaction conditions, using the magnetic acid catalyst WO_3_/CuFe_2_O_4_, was evaluated for its physicochemical properties and compared with the ASTM D6751 international standard. The results are given in Table 2. The kinematic viscosity and density are important fuel properties because the first one shows the ability of a material to flow, and both are related to the quality of fuel atomization and biodiesel's molecular structure.^13^ The biodiesel exhibited kinematic viscosity and density values of 4.7 mm^2^ s^−1^ and 0.881 g cm^−3^, respectively. Based on these results, the biodiesel obtained in this research showed values within limits established by ASTM standard range. The estimated acid value of the synthesized biodiesel was 0.21 mg KOH g^−1^. The low acid value is within the limit defined by ASTM as well as it means that any corrosion will be caused in engine by biodiesel.^55^ The flash point (FP) is another essential fuel property which is an indirect measure of fuel volatility.^56^ The FP measure of biodiesel reached 155 °C, indicating security for storage and portability. The cold filter plugging point (CFPP) is a parameter used to determine the minimum temperature at which fuel filters clog in automotive engines due to partial solidification of fuel.^13^ The biodiesel showed CFPP of 0 °C, inferring that the biodiesel could be used in cold weather countries. In the corrosiveness to copper analysis, the biodiesel presented a value of 1a, suggesting that the biofuel will not cause damage to the engine's metallic components. Similar value was obtained by Gonçalves *et al.*^20^ All results confirm that the waste cooking oil has been successfully converted into biodiesel using WO_3_/CuFe_2_O_4_ magnetic acid catalyst and conform to ASTM D6751 standard. The biodiesel oxidative stability value of 4.80 h is greater than the minimum limit of 3 h defined by ASTM D6751.

**Table 2:** Physicochemical properties of biodiesel produced by WO_3_/CuFe_2_O_4_ magnetic catalyst and their limits^a^

Biodiesel properties	Unit	Test methods	ASTM D6751 limits	Present study
Kinematic viscosity (at 40 °C)	mm^2^ s^−1^	D445	1.9–6.0	4.7
Density (at 20 °C)	g cm^−3^	D6890	0.875–0.900	0.881
Acid value	mg KOH g^−1^	D664	0.5 max	0.21
Flash point	°C	D93	130 min	155
Cold filter plugging point	°C	D6371	NS	0
Copper strip corrosion	—	D130	3 max	1a
Oxidative stability	h	EN 14112	3 min	4.8

a NS = not specified.

### 3.4. Assessment of magnetic catalyst stability WO_3_/CuFe_2_O_4_

**Fig. 10:**
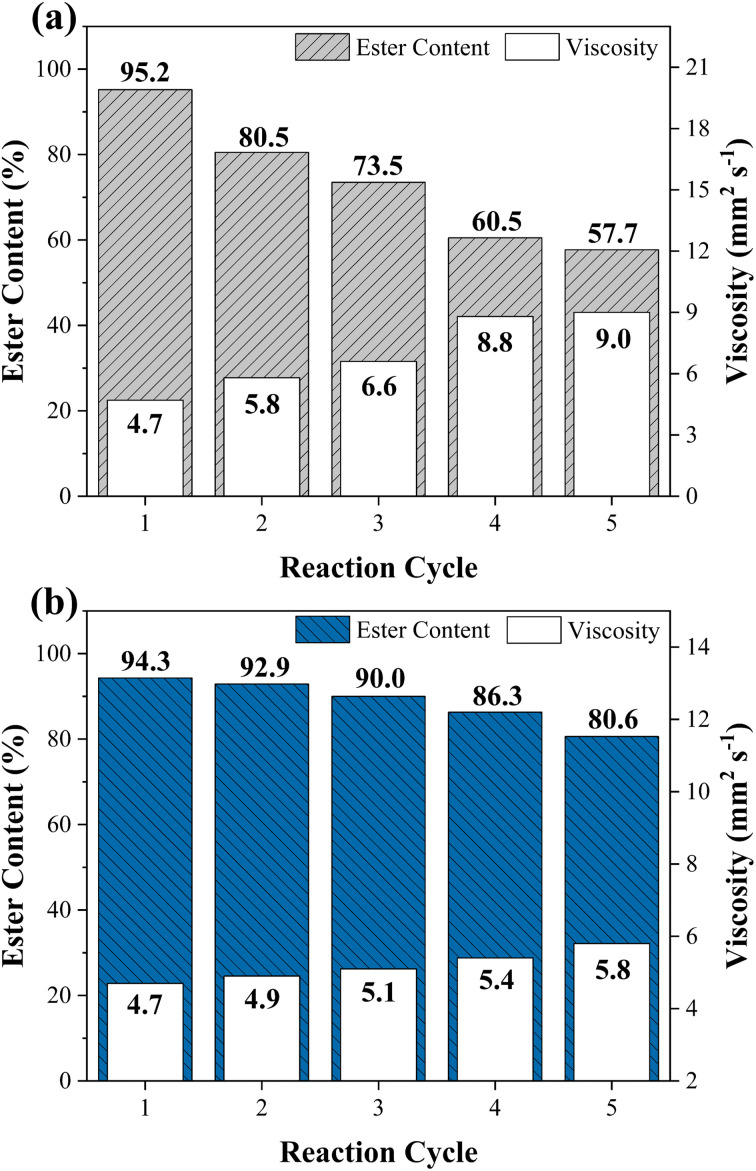
Reuse study of WO_3_/CuFe_2_O_4_ magnetic catalyst in the transesterification reaction (a) with heat treatment and (b) without heat treatment at 500 °C/3 h.

**Fig. 11:**
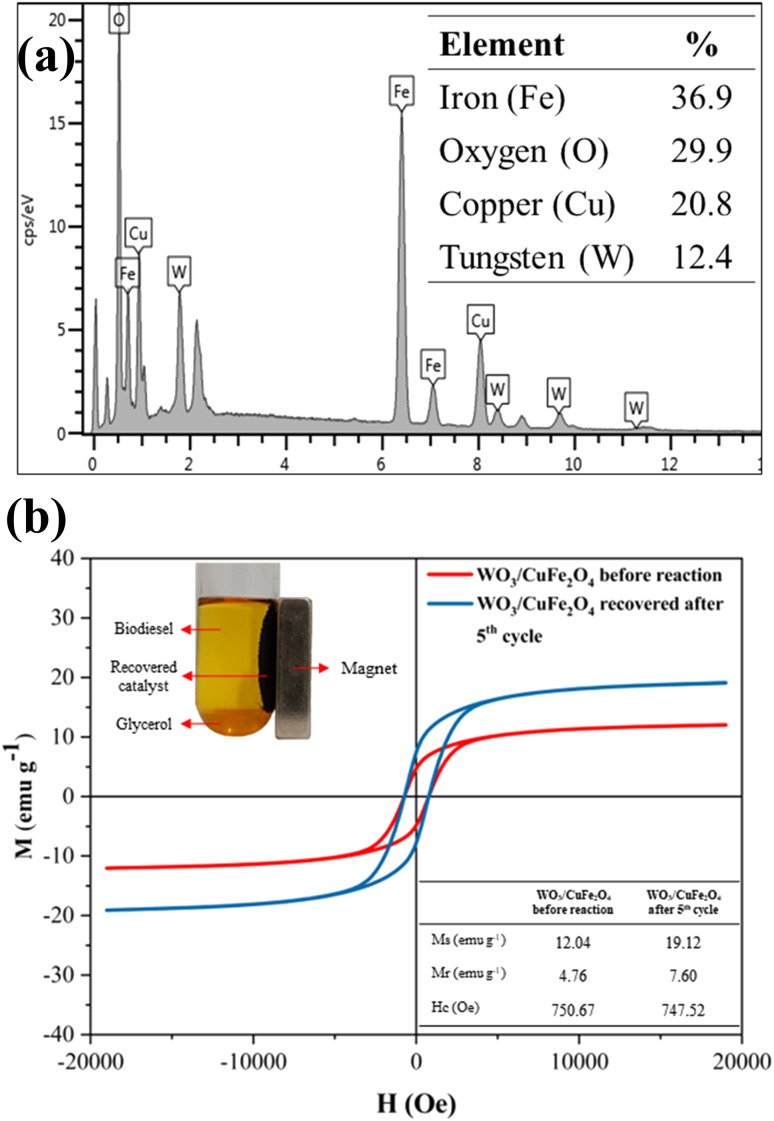
Analysis of (a) EDS and (b) VSM of the WO_3_/CuFe_2_O_4_ catalyst recovered after the 5th reaction cycle.

The reuse and recovery capacity are characteristics that make the heterogeneous catalyst more economically feasible for the biodiesel production process.^55^ The magnetic catalyst WO_3_/CuFe_2_O_4_ was evaluated by carrying out several reaction cycles under the optimal condition of transesterification reaction, as shown in Fig. 10. It is noteworthy that after each reaction cycle, the catalyst was recovered by applying an external magnet, washed with heptane and ethanol to eliminate possible impurities from the catalyst surface from the reaction mixture, and dried in an oven for 12 h. Fig. 10a shows the results obtained in terms of ester content and kinematic viscosity for the synthesized biodiesel. The WO_3_/CuFe_2_O_4_ magnetic catalyst was reused for five reaction cycles and showed a reduction of its catalytic efficiency of approximately 40%. Two reasons may be related to the loss of catalytic activity: (1) partial leaching of the active sites and (2) deposition of organic matter on the catalyst surface. The first hypothesis was verified by analyzing the EDS of the catalyst recovered after the fifth reaction cycle (Fig. 11a), the analysis revealed a decrease in the tungsten concentration (W) present in the catalyst from 35.4% to 12.4%. In addition, there was a reduction in surface acidity value from 7.43 mmol H^+^ g^−1^ (catalyst before reaction) to 3.38 mmol H^+^ g^−1^ (catalyst after the fifth reaction allotment cycle), confirming the leaching of tungsten species from the magnetic catalyst surface.

Despite the decrease in catalytic efficiency of the WO_3_/CuFe_2_O_4_ material during transesterification cycles, the catalyst presented ester content values of 95.2 and 57.7% for the first and fifth reaction cycles, respectively. These values are above the values of ester contents of the biodiesel obtained in the reaction performed without the presence of catalyst (18.7%), and only in the presence of the CuFe_2_O_4_ support (21.4%), indicating efficiency of the active component WO_3_ for the biodiesel production process. These results are correlated with the Surface Acidity of the CuFe_2_O_4_ support (2.71 mmol H^+^ g^−1^) and the catalyst WO_3_/CuFe_2_O_4_ (7.43 mmol H^+^ g^−1^). The intrinsic acidity of CuFe_2_O_4_ was not sufficient to promote the transesterification process. The impregnation with tungsten increased the surface acidity value through the new Brønsted acid sites present in the catalyst, which enable the catalytic activity of the catalyst by its interaction with the carbonyl group in the transesterification process.^27^ Catalyst recovery was 88.05 ± 2.92%, which proves the efficiency of the magnetic separation process employed. Furthermore, the VSM analysis of the catalyst before and after the fifth reaction cycle is displayed in Fig. 11b. When comparing the magnetic characteristics of the materials, it is possible to observe an increase in the value of saturation magnetization (*M*_s_) from 12.04 emu g^−1^ to 19.12 emu g^−1^. This behavior possibly occurs due to leaching of the non-magnetic component (WO_3_) of the magnetic support surface (CuFe_2_O_4_) during reaction cycles, which makes the *M*_s_ value of the reused catalyst closer to the *M*_s_ value of the copper ferrite (22.70 emu g^−1^).

In view of the previous statements, the strategy adopted to improve the catalytic efficiency of the WO_3_/CuFe_2_O_4_ catalyst was to perform a heat treatment by calcination at 500 °C/3 h of the catalyst after each reaction cycle. The reuse study of the catalyst using heat treatment is presented in Fig. 10b and demonstrated superior reuse capacity when compared to the previous results, providing a biodiesel with an 80.6% ester content in the fifth reaction cycle. At first, the calcination catalyst reactivation process is a way to eliminate organic components from the catalyst surface that were not removed in the washing stage, causing a reduction in the efficiency of catalytic solids due to the blocking of active sites, which hinders the access of reagents to these sites.^57^ In addition, heat treatment played a key role in assigning greater stability and maintenance of active species in the magnetic support.

The study of the oxidative stability of the biodiesel produced during the transesterification reaction cycles using WO_3_/CuFe_2_O_4_ was carried out to evaluate the impact of leached species in the oxidation process of the obtained esters (Table S1†). The results of biodiesel's oxidative stability showed values of 4.80, 4.61, 4.17, 3.89 and 3.33 h for the first to the fifth reaction cycle, respectively. The decrease in induction time values implies that leached metals can affect the oxidation process of the obtained samples. However, leaching does not affect the quality of biodiesel obtained through catalyst reuse cycles, because all results are in accordance with the minimum limit of 3 h stipulated by the method ASTM D6751.

The catalytic efficiency and stability of the WO_3_/CuFe_2_O_4_ catalyst are compared with various acid catalysts under the optimal transesterification reaction conditions. The magnetic acid catalyst developed in this study allowed high activity and stability throughout the reactional cycles of transesterification, without significant loss of its catalytic efficiency after five uses. From the analysis of the data contained in Table 3, it is verified that most acid catalysts require higher temperatures and reaction times in order to lead to biodiesel with high ester contents. On the other hand, some works developed exhibit more severe conditions of alcohol : oil molar ratio e catalyst loading.^20,49,60,61^ However, the application of WO_3_/CuFe_2_O_4_ catalyst in the transesterification reaction promoted the production of a biodiesel with an ester content greater than 95%, using milder reaction parameters than most catalysts presented in Table 3 or with similar trend. Therefore, the WO_3_/CuFe_2_O_4_ magnetic acid catalyst can be considered an appropriate choice for the biodiesel production process *via* transesterification.

**Table 3:** Comparison of catalytic performance of different heterogeneous acid catalysts for biodiesel production

Catalyst	Oil feedstocks	Transesterification parameters				Ester content (%)	Cycles	Ref
		Temperature (°C)	Time (h)	Alcohol : oil molar ratio	Catalyst loading (%)			
WO_3_/SnO_2_	Soybean	110	5	30 : 1	5	79.2	5	58
WO_3_/AlPO_4_	Soybean	180	5	30 : 1	5	72.5	5	23
WO_3_/Zr-MCM-41	Sunflower	200	2.5	12 : 1	13.3	92.0	3	48
WO_3_/ZrO_2_	Soybean	200	5	15 : 1	3	97.0	—	59
Nb_2_O_5_/SO_4_	Macaw palm oil	250	4	120 : 1	30	99.2	5	49
HPMo/TiO_2_	Waste cooking oil	190	4	90 : 1	5	94.5	4	20
HPMo/Al_2_O_3_	Macaw palm oil	190	4	50 : 1	13	99.8	4	60
HPMo/Nb_2_O_5_	Macaw palm oil	210	4	90 : 1	20	99.6	4	61
Cs_2.5_PW_12_O_40_	Used vegetable oil	260	0.67	40 : 1	3	92.0	—	62
WO_3_/CuFe_2_O_4_	Waste cooking oil	180	3	45 : 1	6	95.2	5	This study

## 4. Conclusions

The present work evaluated the application of a new heterogeneous magnetic acid catalyst in the transesterification of the waste cooking oil in methyl biodiesel. The WO_3_/CuFe_2_O_4_ catalyst was prepared by wet impregnation of the active phase on the CuFe_2_O_4_ support, synthesized by the coprecipitation method. The analyses of XRD, FTIR, SEM, EDS, TG/DTG, VSM and Surface acidity indicated that the heterogeneous catalyst was successfully formed and confirmed its bifunctional character (catalytic and magnetic activity). The magnetic catalyst developed in this study obtained a biodiesel with an ester content of 95.2% under the optimal reaction condition (reaction temperature of 180 °C, reaction time of 3 h, MeOH : oil molar ratio of 45 : 1 and catalyst loading of 6%). The catalyst maintained its catalytic efficiency leading biodiesel ester content above 80% after five reaction cycles and the magnetic property was maintained as well, making separation by magnetic decanting feasible. Thus, WO_3_/CuFe_2_O_4_ catalyst can be considered promising for the biodiesel production process for its excellent catalytic performance, easy separation and good stability.

## Author contributions

Hiarla Cristina Lima dos Santos: conceptualization, methodology, investigation, visualization, formal analysis, writing – original draft. Matheus Arrais Gonçalves: methodology, resources, investigation. Alexandre da Cas Viegas: methodology, investigation. Bruno Apolo Miranda Figueira: methodology, investigation Patrícia Teresa Souza da Luz: methodology, investigation. Geraldo Narciso da Rocha Filho: supervision. Leyvison Rafael Vieira da Conceição: project administration, conceptualization, visualization, supervision, project administration, writing – review & editing.

## Conflicts of interest

The authors declare that they have no known competing financial interests or personal relationships that could have appeared to influence the work reported in this paper.

## Supplementary Material

RA-012-D2RA06923G-s001
