# Supplementary material for: Tungsten oxide supported on copper ferrite: a novel magnetic acid heterogeneous catalyst for biodiesel production from low quality feedstock
Source: RSC Adv. 2022 Dec 2;12(53):34614–26. doi: 10.1039/d2ra06923g (PMC9716347; doi:10.1039/d2ra06923g)
Supplement: RA-012-D2RA06923G-s001 [file RA-012-D2RA06923G-s001.pdf]

Electronic Supplementary Material (ESI) for RSC Advances.
This journal is © The Royal Society of Chemistry 2022

Suporting Information

Tungsten oxide supported on copper ferrite: A novel magnetic acid heterogeneous catalyst for biodiesel production from low quality feedstock

Hiarla Cristina Lima dos Santos[a], Matheus Arrais Gonçalves[a], Alexandre da Cas Viegas[b], Bruno Apolo Miranda Figueira[c], Patrícia Teresa Souza da Luz[d], Geraldo Narciso da Rocha Filho[a], Leyvison Rafael Vieira da Conceição[a*].

[a] Federal University of Pará, Institute of Exact and Natural Sciences, Graduate in Chemistry Program, Laboratory of Catalysis and Oleochemical, 66075–110, Belém, Pará, Brazil.

[b] Federal University of Rio Grande do Sul, Institute of Physics, 90035–190, Porto Alegre, Rio Grande do Sul, Brazil.

[c] Federal University Western Pará, Graduate in Environmental Society and Quality of Life, 68040–255, Santarém, Pará, Brazil.

[d] Federal Institute of Education, Science and Technology of Pará, Department of Education, Science and Teacher Education, 66093–020, Belém, Pará, Brazil.

*Autor correspondente. Tel.: +55 91 98102 1185.

E-mail address: rafaelvieira@ufpa.br (Leyvison Rafael Vieira da Conceição).

Contents

1. WO_3 loading study ....Figure S1
2. Oxidative stability results of the biodiesels ....Table S1

1. WO_3 loading study

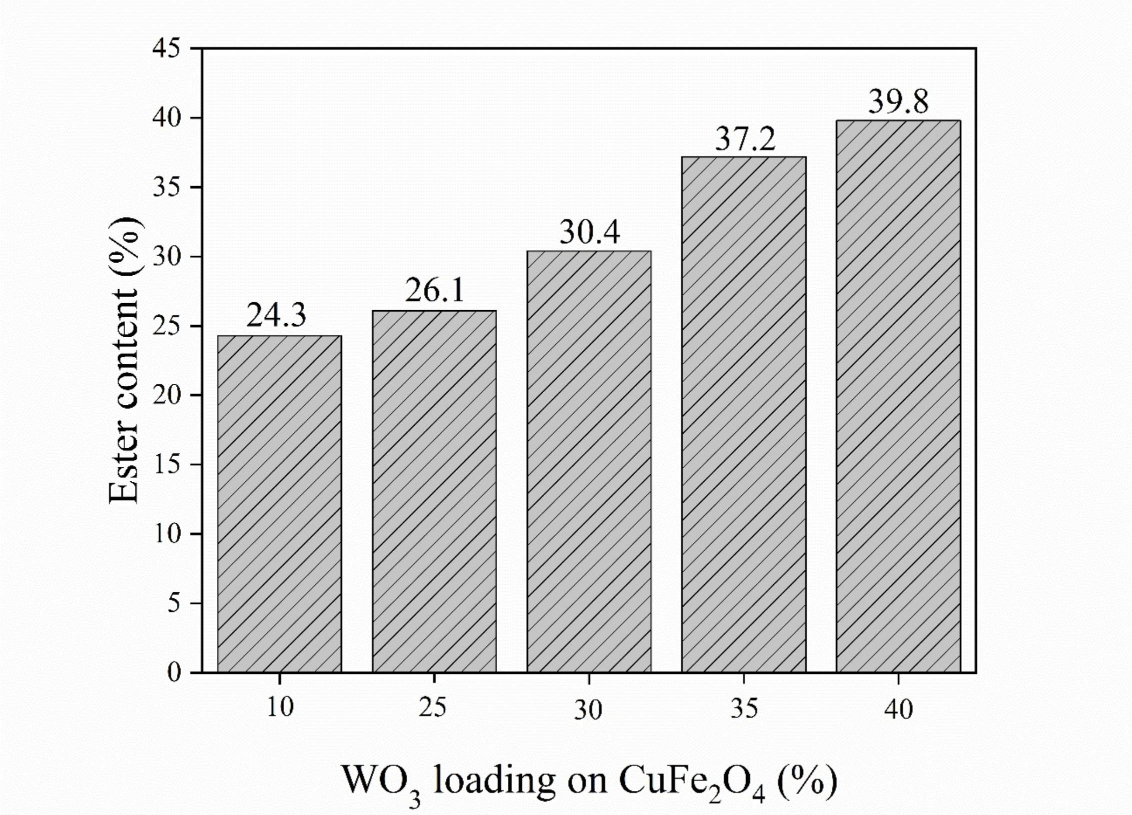

Fig. S1 Assessment of WO_3 loading on $CuFe_2O_4$ magnetic support in transesterification reaction (reaction temperatura of 160 ºC, reaction time of 3 h, MeOH:oil molar ratio of 35:1, catalyst loading of 6%).

2. Oxidative stability results of the biodiesel

Table S1 Oxidative stability results of the biodiesels obtained in the reaction cycles using $WO_3/CuFe_2O_4$ magnetic catalyst.

**Reaction cycles**	**Ester content (%)**	**Oxidative stability (h)**
**1**	94.3	4.80
2	92.9	4.61
3	90.0	4.17
4	86.3	3.89
5	80.6	3.33
